# Supplementary material for: Genome-Wide Association Study Identifies Candidate Loci Associated with Opioid Analgesic Requirements in the Treatment of Cancer Pain
Source: Cancers (Basel). 2022 Sep 27;14(19):4692. doi: 10.3390/cancers14194692 (PMC9564079; doi:10.3390/cancers14194692)
Supplement: Supplementary file 1 [file cancers-14-04692-s001.zip › cancers-1891486-supplementary.pdf]

Supplementary Materials

Genome-wide association study identifies candidate loci associated with opioid analgesic requirements in the treatment of cancer pain

Daisuke Nishizawa [1], Takeshi Terui [2], Kunihiko Ishitani [2], Shinya Kasai [1], Junko Hasegawa [1], Kyoko Nakayama [1], Yuko Ebata [1], Kazutaka Ikeda [1,*]

Supplementary Table S1. Estimated systemic dose equipotent to 90 mg oral morphine (mg).

Analgesics	Dose
Morphine (oral)	90
Morphine (intravenous, subcutaneous)	30
Oxycodone (oral)	60
Oxycodone (intravenous, subcutaneous)	45
Fentanyl (intravenous)	0.9
Fentanyl (transdermal)	3
Tapentadol	300
Tramadol	450
Methadone (oral)	18
Hydromorphone (oral)	18
Hydromorphone (intravenous)	3.6

Supplementary Table S2. Top 10 candidate gene sets selected from gene-set analysis.

Model, genetic model in which candidate gene sets were selected by analysis; nGenes, number of genes in the data that are in the gene set; Beta, regression coefficient of the gene set; SE, standard error of the regression coefficient; p^a, adjusted p value for multiple testing.

Model	Rank	Gene set name	nGenes	Beta	SE	*p*	p^a
Additive	1	go_arginine_binding	7	1.5697	0.36089	0.106308293	0.106308293
Additive	2	go_sleep	31	0.61055	0.15283	0.50337243	0.50337243
Additive	3	boyault_liver_cancer_subclass_g6_up	66	0.43693	0.10985	0.541328616	0.541328616
Additive	4	reactome_interleukin_37_signaling	20	0.74948	0.19082	0.667012992	0.667012992
Additive	5	go_negative_regulation_of_torc1_signaling	14	0.95642	0.24846	0.920240064	0.920240064
Additive	6	watanabe_ulcerative_colitis_with_cancer_dn	15	0.78085	0.20622	1.187884602	1
Additive	7	go_rrna_adenine_methyltransferase_activity	8	0.91368	0.25542	2.69750634	1
Additive	8	biocarta_fas_pathway	30	0.54087	0.15477	3.68427426	1
Additive	9	go_primitive_hemopoiesis	8	1.0388	0.29812	3.8304621	1
Additive	10	go_positive_regulation_of_extrinsic_apoptotic_signaling_pathway_via_death_domain_receptors	17	0.66703	0.19962	6.4669536	1
Dominant	1	go_phosphatidylglycerol_biosynthetic_process	7	1.0318	0.27705	0.000098374	1
Dominant	2	wong_embryonic_stem_cell_core	322	0.15449	0.043806	0.00021101	1
Dominant	3	gentile_uv_response_cluster_d9	26	0.61884	0.1782	0.00025822	1
Dominant	4	go_membrane_hyperpolarization	11	0.82733	0.25692	0.00064192	1
Dominant	5	reactome_rrna_modification_in_the_mitochondrion	6	0.93826	0.29291	0.00068067	1
Dominant	6	go_axonal_transport_of_mitochondrion	10	0.73055	0.22977	0.00073917	1
Dominant	7	go_clathrin_sculpted_gamma_aminobutyric_acid_transport_vesicle_membrane	8	0.89238	0.28304	0.00081001	1
Dominant	8	cadwell_atg16l1_targets_up	96	0.25946	0.082733	0.0008576	1
Dominant	9	go_response_to_tumor_necrosis_factor	288	0.1631	0.052997	0.0010456	1
Dominant	10	go_negative_regulation_of_epithelial_cell_migration	63	0.33269	0.10891	0.0011278	1
Recessive	1	go_arginine_binding	7	1.5647	0.36193	7.7371E-06	0.119793519
Recessive	2	go_sleep	31	0.65245	0.15327	0.000010422	0.161363826
Recessive	3	reactome_cytosolic_iron_sulfur_cluster_assembly	12	1.3065	0.31084	0.000013248	0.205118784
Recessive	4	go_mmxd_complex	4	1.8154	0.43512	0.000015171	0.234892593
Recessive	5	go_cia_complex	5	1.6469	0.41628	0.000038244	0.592131852
Recessive	6	boyault_liver_cancer_subclass_g6_up	64	0.43122	0.11171	0.000056909	0.881122047
Recessive	7	reactome_interleukin_37_signaling	20	0.73232	0.19138	0.000065252	1
Recessive	8	ingram_shh_targets_dn	56	0.45578	0.11956	0.000069166	1
Recessive	9	go_regulation_of_triglyceride_metabolic_process	8	1.1279	0.30386	0.00010326	1
Recessive	10	go_negative_regulation_of_torc1_signaling	14	0.91368	0.24919	0.00012332	1

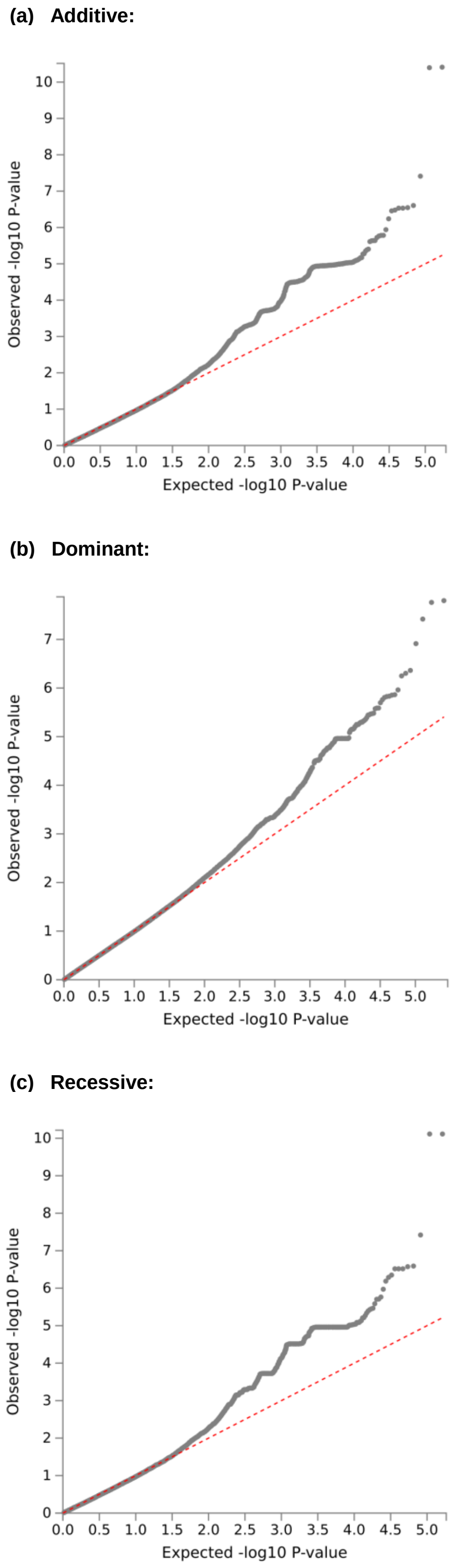

Figure S1. Log quantile-quantile (QQ) p-value plot as a result of the GWAS for all patient samples. (a) Plot of the results from the additive model. (b) Plot of the results from the dominant model. (c) Plot of the results from the recessive model.

(a) Additive:

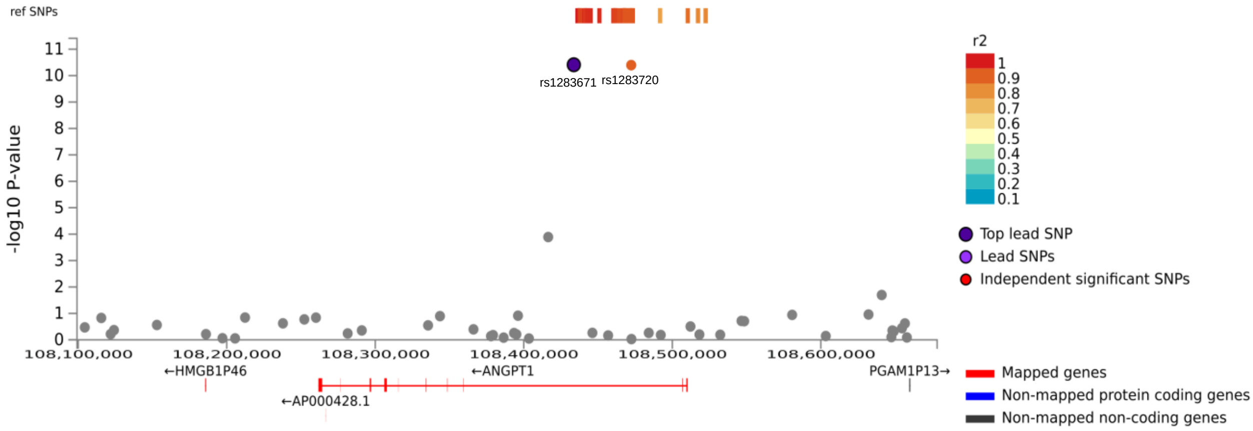

(b) Dominant:

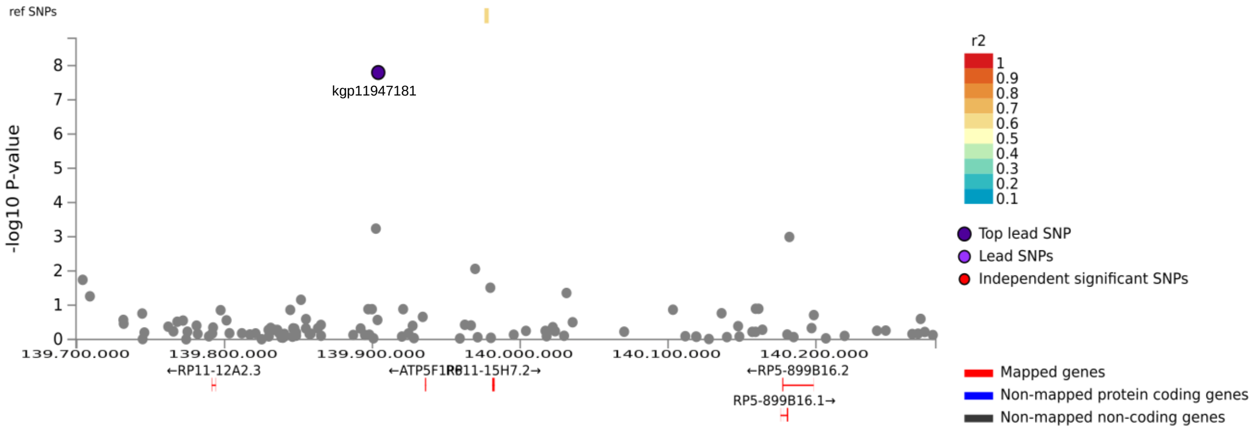

(c) Recessive:

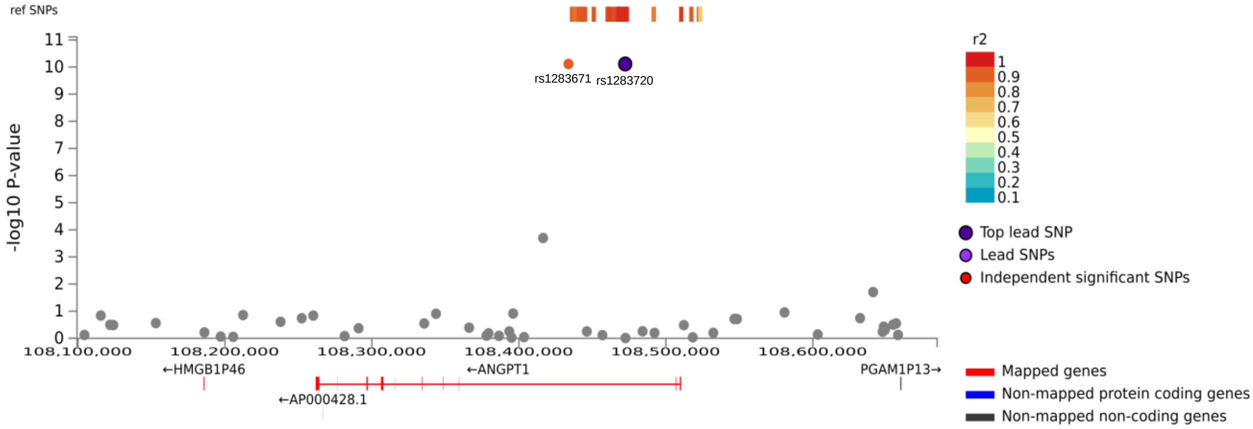

Figure S2. Regional plots of potent loci that were associated with daily opioid requirements in cancer pain treatment. The results of the association analyses in each genetic model were plotted, with information on annotated genes, referenced SNPs in the database (ref SNPs), and the pairwise-calculated strength of linkage disequilibrium (LD; r^2 values) with the top lead SNP in these regions. (a) The genomic position from ~108 100 000 to ~108 670 000 on chromosome 8 (GRCh37) is illustrated with the results of the association analysis in the additive model. (b) The genomic position from ~139 700 000 to ~140 270 000 on chromosome 6 (GRCh37) is illustrated with the results of the association analysis in the dominant model. (c) The genomic position from ~108 100 000 to ~108 670 000 on chromosome 8 (GRCh37) is illustrated with the results of the association analysis in the recessive model.

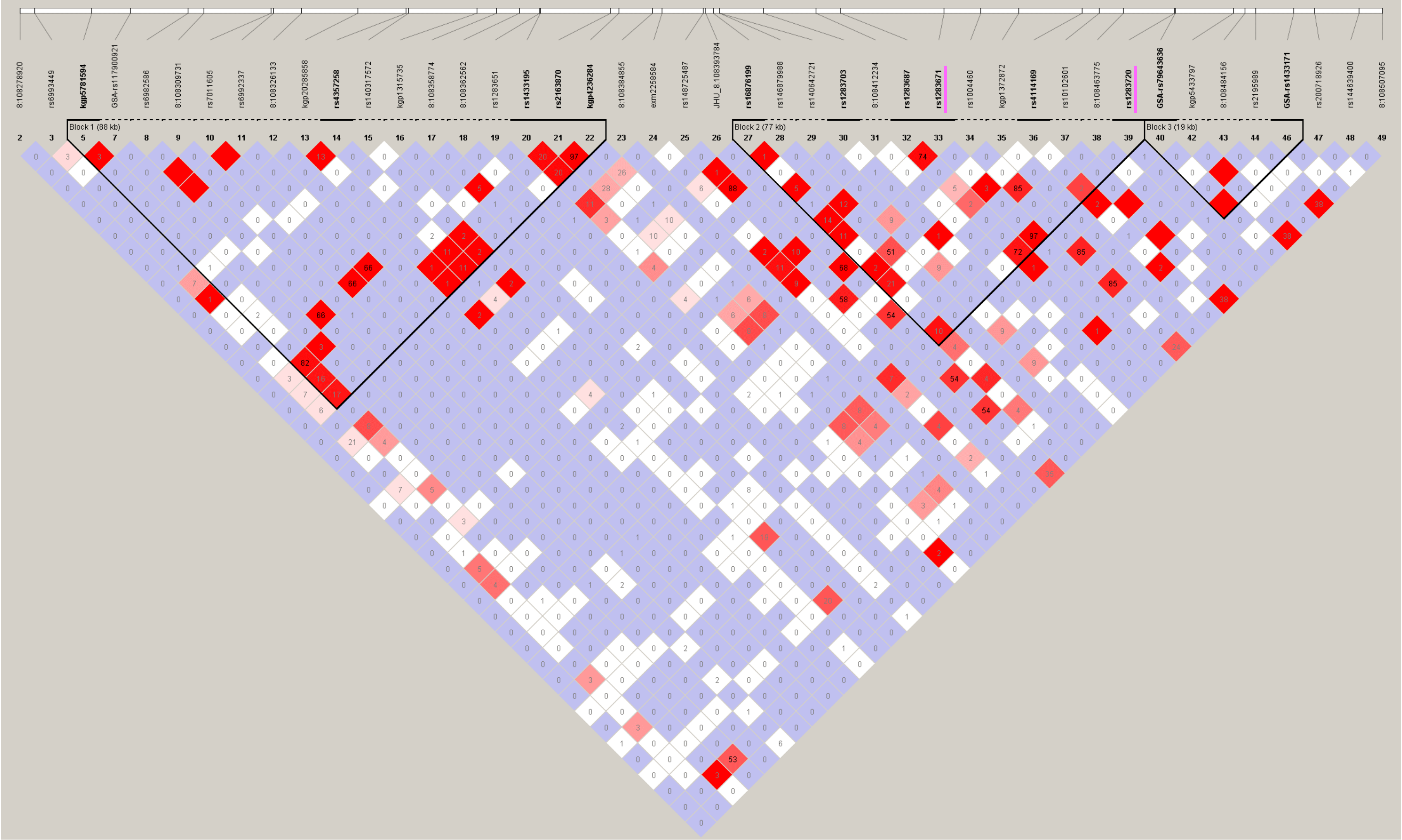

Figure S3. State of linkage disequilibrium (LD) between SNPs in the genomic region of the *ANGPT1* gene. The genotype data for the 428 patient subjects that passed the quality control criteria were used in the LD analysis, and SNPs with a minor allele frequency ≥ 0.05 were selected in the genomic position around the rs1283671 SNP and rs1283720 SNP, which spans from ~170 kbp upstream of the rs1283671 SNP to ~35 kbp downstream of the rs1283720 SNP. Numbers in squares in which two SNPs face each other represent the percentage of r^2 values that were calculated from genotype data of the SNPs. Blank squares represent $r^2 = 1$. The rs1283671 and rs1283720 SNPs are highlighted with pink lines.
